# Sex-specific changes in protein expression of membrane transporters in the brain cortex of 5xFAD mouse model of Alzheimer’s disease

**DOI:** 10.3389/fphar.2024.1365051

**Published:** 2024-03-20

**Authors:** Elena Puris, Liudmila Saveleva, Seppo Auriola, Mikko Gynther, Katja M. Kanninen, Gert Fricker

**Affiliations:** ^1^ Institute of Pharmacy and Molecular Biotechnology, Ruprecht-Karls-University, Heidelberg, Germany; ^2^ A.I. Virtanen Institute for Molecular Sciences, University of Eastern Finland, Kuopio, Finland; ^3^ School of Pharmacy, University of Eastern Finland, Kuopio, Finland

**Keywords:** Alzheimer’s disease, membrane transporters, brain, 5XFAD mice, animal model, sex, age

## Abstract

Membrane transporters playing an important role in the passage of drugs, metabolites and nutrients across the membranes of the brain cells have been shown to be involved in pathogenesis of Alzheimer’s disease (AD). However, little is known about sex-specific changes in transporter protein expression at the brain in AD. Here, we investigated sex-specific alterations in protein expression of three ATP-binding cassette (ABC) and five solute carriers (SLC) transporters in the prefrontal cortex of a commonly used model of familial AD (FAD), 5xFAD mice. Sensitive liquid chromatography tandem mass spectrometry-based quantitative targeted absolute proteomic analysis was applied for absolute quantification of transporter protein expression. We compared the changes in transporter protein expressions in 7-month-old male and female 5xFAD mice versus sex-matched wild-type mice. The study revealed a significant sex-specific increase in protein expression of ABCC1 (*p* = 0.007) only in male 5xFAD mice as compared to sex-matched wild-type animals. In addition, the increased protein expression of glucose transporter 1 (*p* = 0.01), 4F2 cell-surface antigen heavy chain (*p* = 0.01) and long-chain fatty acid transport protein 1 (*p* = 0.02) were found only in female 5xFAD mice as compared to sex-matched wild-type animals. Finally, protein expression of alanine/serine/cysteine/threonine transporter 1 was upregulated in both male (*p* = 0.02) and female (*p* = 0.002) 5xFAD mice. The study provides important information about sex-specific changes in brain cortical transporter expression in 5xFAD mice, which will facilitate drug development of therapeutic strategies for AD targeting these transporters and drug delivery research.

## 1 Introduction

Alzheimer’s disease (AD) remains the leading form of dementia worldwide (Cahill, 2020). AD is not a normal part of aging (Varga-Medveczky et al., 2021; Lau et al., 2023). The major hallmarks of AD are represented by formation of the brain extracellular amyloid β (Aβ) plaques together with intracellular accumulation of hyperphosphorylated tau in neurofibrillary tangles (NFTs), resulting in synaptic impairment and inflammation (Scheltens et al., 2021). Due to the lack of intervention strategies, there is an unmet need for development of disease-modifying therapies to combat AD.

The development of effective drugs requires reliable translation from preclinical data to clinical settings, which depends on selection of a relevant animal model to be used. Various animal models of AD have been developed to investigate the pathogenesis of the disease and test novel drug candidates (Jankowsky and Zheng, 2017; McKean et al., 2021). The majority of the transgenic AD models such as 3xTg, APP/PS1, 5xFAD, PS2APP, Tg2576, TgCRND8, TAPP, are generated to mimic genetic mutations in amyloid precursor protein (APP) and/or presenilins (PSEN) relevant to familial AD (FAD) (Yokoyama et al., 2022). FAD is a much less common form of AD compared to a late-onset AD (LOAD) with symptoms arising after the age of 65 years (van der Flier et al., 2011; Hinz and Geschwind, 2017). However, only limited models of LOAD variants including Apolipoprotein E (APOE) and TREM2 have been developed (Holtzman et al., 2000; Jay et al., 2017). Thus, the animal models of FAD remain the major preclinical tools in AD drug development. However, FAD models do not fully represent LOAD, rather they recapitulate specific pathological features, such as, for instance, Aβ pathology. Therefore, a thorough evaluation of each individual animal model is necessary to prove the relevance to replicate AD effects because the degree of characteristics revealed in AD transgenic animals is constrained in comparison to AD patients. Additionally, women, who represent two-thirds of AD patients, showed faster cognitive decline after a diagnosis of AD dementia than men (Gao et al., 1998; Ferretti et al., 2018; Laws et al., 2018). Therefore, choosing amongst the animal models necessitates knowing the exact characteristics modeled and taking sex into account given the existence of sex-related differences in the underlying pathophysiology of AD (Filon et al., 2016).

Recent studies provided evidence that transporters, such as Solute carrier transporters (SLCs) and adenosine triphosphate (ATP)-binding cassette transporters (ABCs), expressed at the membranes of the neurovascular unit (NVU) cells (microvascular endothelial cells, glial cells, pericytes) play potential roles in the development and progression of AD (Wolf et al., 2012; Pahnke et al., 2014; Pereira et al., 2018; Jia et al., 2020). The transporters expressed at the cells of the NVU mediate uptake and efflux of solutes, such as nutrients, metabolites and drugs, across the membranes of the NVU cells including the blood-brain barrier (BBB) (Abbott et al., 2010). Thus, the altered SLC and ABC transporters’ expression and function can be a reason for biochemical perturbations observed in AD brains as well as influence drug delivery to the brain and within the brain resulting in altered treatment efficacy. ABC transporters have been shown to be involved in efflux of Aβ from the brain across the BBB (Wolf et al., 2012; Pahnke et al., 2014), which resulted in the hypothesis that Aβ accumulation in the brain may be due to reduced clearance via ABC transporters. In previous quantitative studies by us and others, significant alterations in protein expression of ABC and SLC transporters in the isolated brain microvessels and brain cortical tissue in animal models and AD patients were found (Puris et al., 2022a; Puris et al., 2022c; Puris et al., 2023). Moreover, we reported sex-specific changes in expression of ATP-binding cassette subfamily C member 1 (ABCC1), and 4F2 cell-surface antigen heavy chain (4F2hc) transporters in TgF344-AD rats as compared to sex-matched wild-type (WT) animals (Puris et al., 2022a). As transporters are key players in the passage of nutrients, drugs and metabolites, the investigation of sex discrepancies in transporter expression alterations in AD animal models is crucial for understanding the mechanisms underlying AD pathology, use of relevant models for drug testing, translating preclinical data to humans as well as development of efficient strategies to deliver drugs to the brain.

In the present study, we investigated sex-related changes in transporter protein expression in the brain prefrontal cortex of a commonly used model of FAD, the transgenic 5xFAD mouse model. The 5xFAD mouse model is designed to reproduce FAD by expressing human APP and PSEN1 transgenes with five mutations [APP I716V (Florida), APP KM670/671NL (Swedish), APP V717I (London), PSEN1 L286V, PSEN1 M146L] linked to FAD (Oakley et al., 2006). At 7 months old, 5xFAD mice possess several important pathological features of AD such as a rapid plaque development of amyloid plaque pathology, astrogliosis and microgliosis proportional to amyloid deposition, neurodegeneration, neuronal loss, deficits in synaptic transmission, spatial memory deficits and cognitive impairment (Oakley et al., 2006; Forner et al., 2021; Shin et al., 2021). Here, we quantified and compared protein expression levels of three ABC and five SLC transporters in the cortices of 7-month-old male 5xFAD mice versus sex-matched WT controls using previously developed sensitive liquid chromatography with tandem mass spectrometry (LC–MS/MS)-based quantitative targeted absolute proteomics methods (Puris et al., 2022a; Puris et al., 2023). The changes in absolute protein expression of the transporters were then compared to those in the brain cortical tissue of 7-month-old female mice, which we previously reported (Puris et al., 2022c) in order to identify sex-specific alterations in transporter expression in 5xFAD mice.

## 2 Materials and methods

### 2.1 Animals and tissue collection

The animal study was performed according to the principles and procedures outlined in the Council of Europe Legislation and Regulation for Animal Protection, the ARRIVE guidelines and EU Directive 2010/63/EU for animal experiments. The animal experiments were approved by the Animal Experiment Board in Finland, Regional State Administrative Agency of Southern Finland (ESAVI-2018-012856). Transgenic male and female hemizygous 5xFAD mice (RRID:MMRRC_034848-JAX, Jackson Laboratories, Bar Harbor, ME, United States) and their wild-type (WT) littermates were maintained on the C57BL/6J background (RRID:IMSR_JAX:000664, Jackson Laboratories, Bar Harbor, ME, United States). Transgenic 5xFAD carrying human APP with the APP Swedish, Florida, and London mutations, as well as human PSEN1 with the M146L and L286V mutations, which were driven by the mouse Thy1 promoter were used in the study (Jawhar et al., 2012). The genotyping was performed according to the procedure described previously (Puris et al., 2023). All mice were 7-month-old males (*n* = 8). In addition, samples from 7-month-old female 5xFAD and WT mice (*n* = 8–9) from our previous study were used (Puris et al., 2022c). The mice were housed in plastic cages (*n* = 3–5 mice per cage) under controlled standardized temperature and humidity with 12:12-h light-dark cycles, and free access to water and food. The present study is an exploratory study. In the present study, the sample size for both male WT and 5xFAD mice of 8 (eight) animals per group was based on previous similar studies in mice (*n* = 3–5) (Pan et al., 2018, 2019; Puris et al., 2021) and ASCT1 protein expression level in the prefrontal cortex of 5xFAD mice from our recent study (Puris et al., 2022c). The sample size calculation for the two-sided t-test with 5% significant level and 80% power resulted in seven observations per group.

On the decapitation day, animals were terminally anesthetized with 2,2,2-Tribromoethanol (Sigma-Aldrich, United States), like in the previous study (Puris et al., 2023), to which the results of the current study are compared. To remove the blood, transcardial perfusion with heparinized 0.9% saline (2500 IU/L, LEO) was carried out. Animal brains were removed from the skull followed by extraction of the cerebrums. A part of the prefrontal cortex (ca 15 mg) was snap-frozen and stored at −70°C until further analysis described below.

### 2.2 Quantification of total Aβ_1–42_

Total Aβ_1–42_ levels were quantified in the brain mouse cortical tissues, which were prepared according to the previously described protocol (Puris et al., 2022a). Human Aβ_42_ ELISA Kit (Thermo Fisher Scientific, #KHB3544) was used, and the manufacturer’s instructions were followed. The brain cortical concentration of Aβ_1–42_ was normalized to total protein concentrations measured using Bio-Rad DC Protein Assay (#5000112). The measured brain cortical concentrations of Aβ_1–42_ in the 7-month-old male 5xFAD mice (*n* = 6) were compared to previously reported by us concentrations of Aβ_1–42_ in 7-month-old female 5xFAD mice (Puris et al., 2022c).

### 2.3 Gene expression of inflammation markers

A quantitative real-time polymerase chain reaction (qRT-PCR) was used for quantification of gene expression of inflammation markers, i.e., a marker of microglial activation, allograft inflammatory factor 1 (*Aif1*), a marker of abnormal activation and proliferation of astrocytes, glial fibrillary acidic protein (*Gfap*), and pro-inflammatory cytokine, Interleukin-1 β (*Il1b*) in the brain cortices of 7-month-old male (*n* = 7) and female 5xFAD (*n* = 8) and sex-matched WT mice (*n* = 8). The procedure is described in the Supplementary Material.

### 2.4 Absolute quantification of transporter protein expression

Crude membrane fractions from the brain cortical tissues of 7-month-old male 5xFAD and WT mice were isolated using ProteoExtract^®^ Subcellular Proteome Extraction Kit (#539790, Merck KGaA, Darmstadt, Germany) in accordance with the manufacturer’s instructions. The same procedure and reagents were used for crude membrane isolation from the brain cortical tissues in 7-month-old female 5xFAD and WT mice (Puris et al., 2022c), to which the results of the current study are compared. The crude membrane fractions were stored at −70°C. Total protein levels were measured in the crude membrane fractions using the Bio-Rad DC Protein Assay before the quantitative targeted absolute proteomics analysis.

Absolute protein expression of three ABC transporters, five SLC transporters and a membrane marker, Na^+^/K^+^-ATPase (Supplementary Table S2), were quantified in crude membrane fractions of the brain cortices in 7-month-old male 5xFAD and WT mice (*n* = 8) as described previously (Puris et al., 2022a; Puris et al., 2022c; Puris et al., 2023). The selection of transporters has been made previously (Puris et al., 2022a) by taking into account a potential or verified contribution to AD in order to compare the absolute protein expression levels of the transporters to previously reported ones in 7-month-old female 5xFAD mice and corresponding WT controls (Puris et al., 2022c; Puris et al., 2023). The preparation of peptide samples and LC-MS/MS-based quantitative targeted absolute proteomic analysis were performed as described previously (Uchida et al., 2011; Uchida et al., 2013; Puris et al., 2022a; Puris et al., 2022c; Puris et al., 2023) and described in detail in Supplementary Material. The sample preparation and LC-MS/MS-based quantitative targeted absolute proteomic analysis were performed in a similar way and at the same time for both female (Puris et al., 2022c) and male mice.

### 2.5 Statistical analysis

The data are shown as mean ± standard deviation (SD) for the absolute protein expression levels, normalized fold mRNA expression and total brain cortical concentrations of Aβ_1–42_. To identify the sex-specific changes in transporter expression and inflammation markers, statistical significance of differences in transporter protein expression as well as normalized fold changes in mRNA expression of *Aif1*, *Gfap* and *Il1b* in 5xFAD vs. sex-matched WT mice were analyzed by unpaired t-test for each sex separately. Statistical significance of differences in total brain cortical concentrations of Aβ_1–42_ in male and female 5xFAD mice was analyzed using Mann-Whitney U-test. A *p*-value < 0.05 was considered statistically significant. Data analysis was performed using GraphPad Prism, version 9.0.1 (GraphPad Software, San Diego, CA, United States).

## 3 Results

### 3.1 Aβ pathology and inflammation in 5xFAD mouse model

**FIGURE 1:**
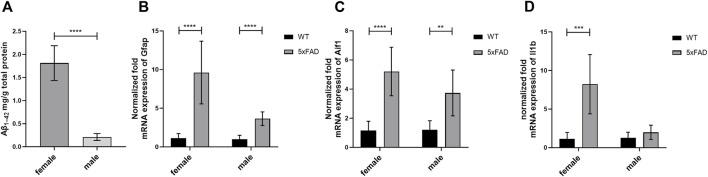
**(A)** The total concentration of brain cortical Aβ_1–42_ in 7-month-old male (*n* = 6) and female (*n* = 8) 5xFAD mice. The data are presented as mean ± SD. Statistical significance of differences between 7-month-old male vs. female 5xFAD mice was analyzed using Mann-Whitney U-test. **(B–D)** Comparison of gene expression of glial fibrillary acidic protein (Gfap), allograft inflammatory factor 1 (Aif1) and Interleukin-1 β (Il1b) in the brain cortical tissue of the 7-month-old male (*n* = 8) and female 5xFAD mice (*n* = 8) vs. age- and sex-matched WT mice (*n* = 8). The gene expression was normalized against the glyceraldehyde-3-phosphate dehydrogenase (Gapdh) house-keeping gene. The data are presented as mean ± SD. Statistical significance of differences between 7-month-old male and female 5xFAD mice vs. corresponding sex-matched wild-type (WT) controls was analyzed using unpaired t-test. Statistically significant differences are marked with asterisks, where **p < 0.01, ***p < 0.005, ****p < 0.0001.

In the present study, we quantified total concentrations of Aβ_1–42_ in the brain cortical tissue of male 5xFAD mice (Figure 1A) and compared the levels to previously reported concentrations in female 5xFAD mice (Puris et al., 2022c). As a result, significantly lower concentrations of Aβ_1–42_ were detected in the brain cortex of male 5xFAD mice compared to female mice (*p* < 0.0001). Similar to our previous reports, Aβ_1–42_ concentrations were under the limit of quantification in the brain cortical tissue of male WT mice (ULQ for Aβ_1–42_ < 1.56 pg/mL).

The normalized mRNA expression of a marker of abnormal activation and proliferation of astrocytes, *Gfap*, was 3.6 and 9.6 times higher in male and female 5xFAD mice (*p* < 0.0001 for both sexes) as compared to sex-matched WT controls, respectively (Figure 1B). Moreover, 3.7- and 5.2-fold increase in normalized fold expression of *Aif1*, a marker of microglial activation, was observed in male (*p* = 0.001) and female 5xFAD mice (*p* < 0.0001) as compared to sex-matched WT controls, respectively (Figure 1C). While no statistically significant differences in normalized mRNA expression of a pro-inflammatory cytokine, *Il1b*, were observed in male groups (Figure 1D), a significant 8.2-fold increase was observed in female 5xFAD mice (*p* = 0.0004) as compared to sex-matched WT controls.

### 3.2 Sex-specific changes in transporter protein expression

**FIGURE 2:**
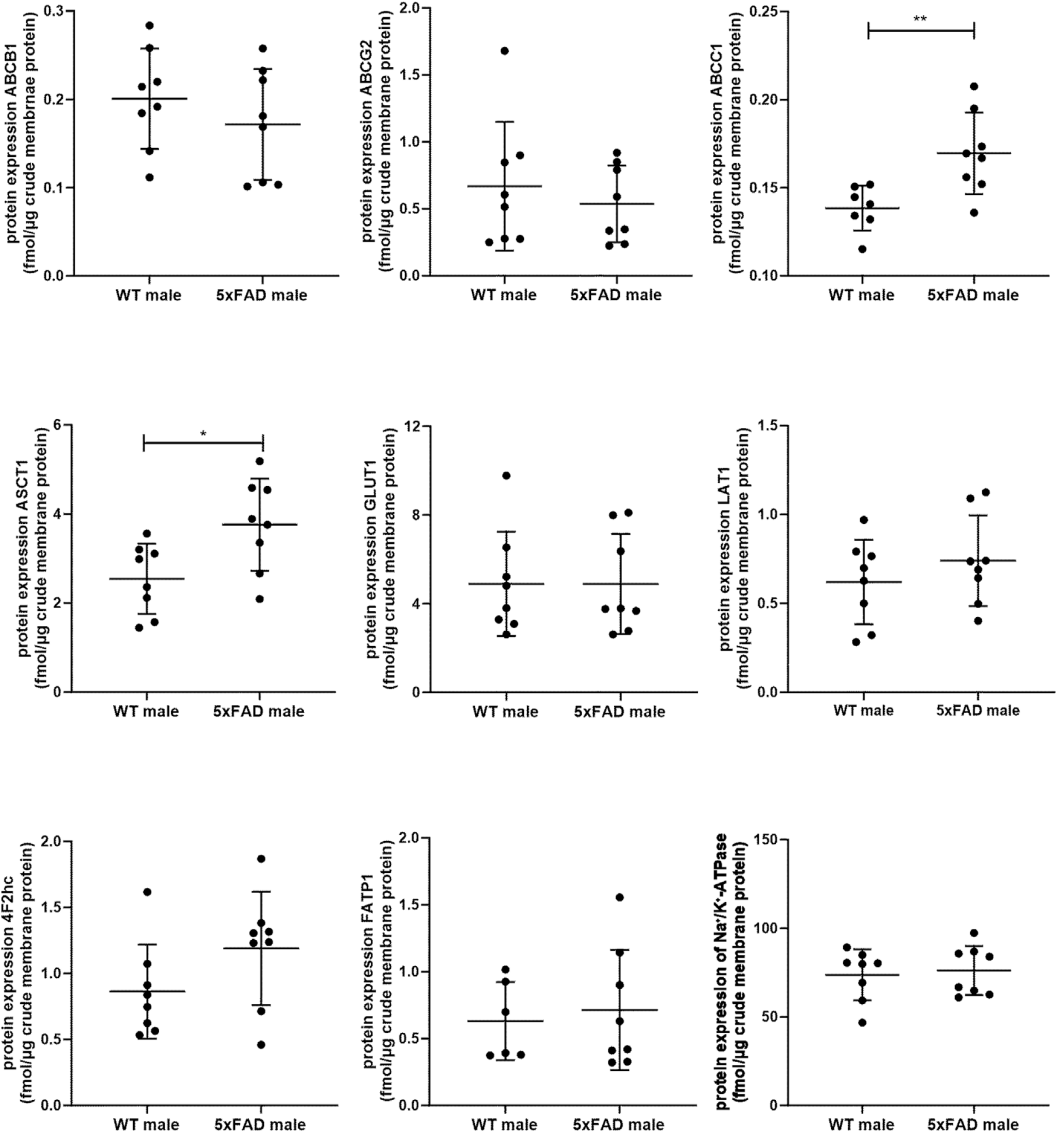
Comparison of absolute protein expression of ABC and SLC transporters (fmol/μg total protein) in crude membrane fraction of the brain cortical tissue in 7-month-old male wild-type (WT) (*n* = 8) and 5xFAD (*n* = 8) mice. Line represents group mean and whiskers the SD. Statistical significance of changes in protein expression between groups was analyzed by unpaired t-test. Statistically significant differences are marked with asterisks, where * indicates *p* < 0.05, **p < 0.01.

**FIGURE 3:**
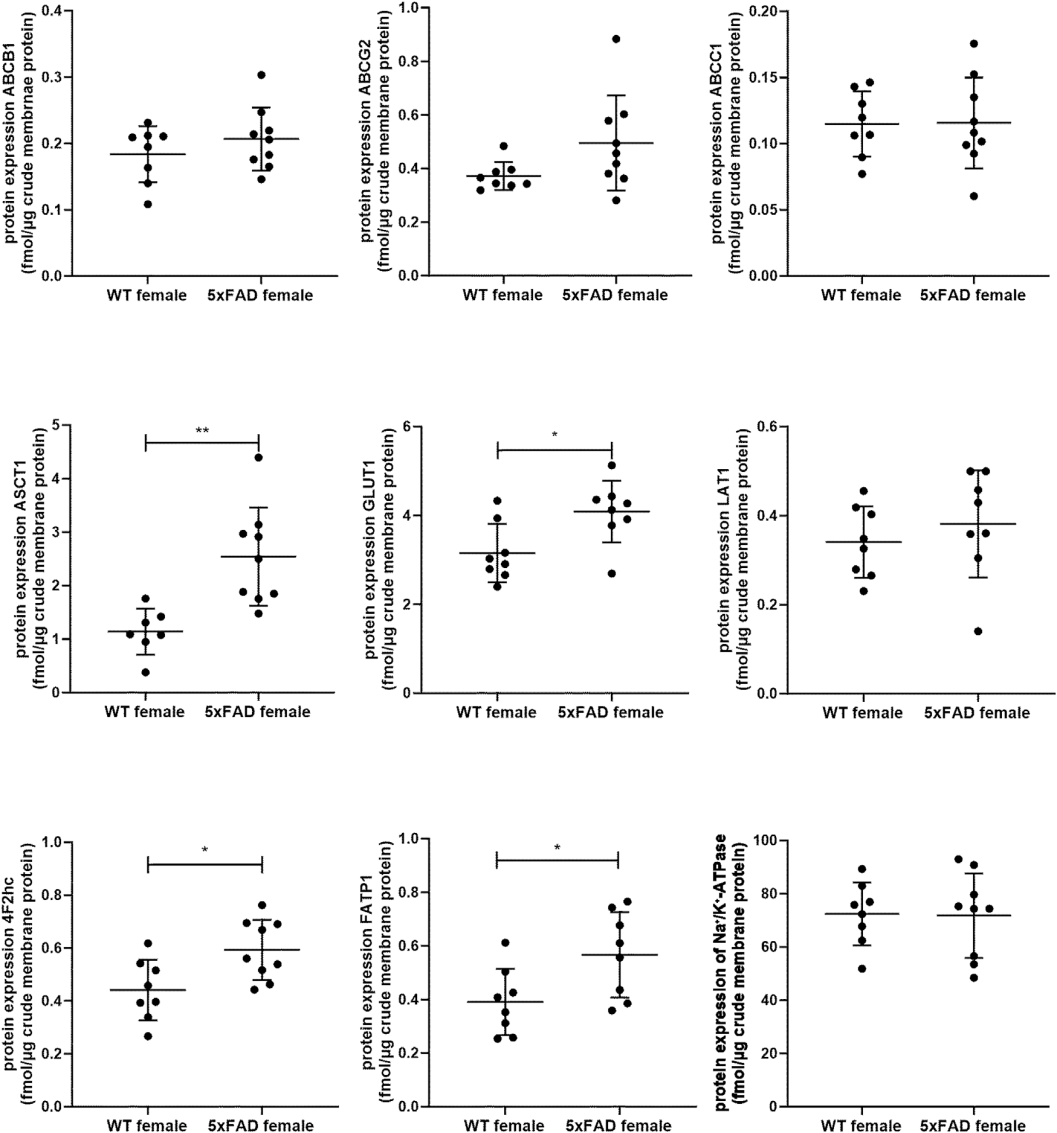
Comparison of absolute protein expression of ABC and SLC transporters (fmol/μg total protein) in crude membrane fraction of the brain cortical tissue in 7-month-old female wild-type (WT) (*n* = 8) and 5xFAD (*n* = 8–9) mice (Puris et al., 2022c). Line represents group mean and whiskers the SD. Statistical significance of changes in protein expression between groups was analyzed by unpaired t-test. Statistically significant differences are marked with asterisks, where * indicates *p* < 0.05, **p < 0.01.

In the present study, we quantified and compared absolute protein expression of three ABC and five SLC transporters in the crude membrane fraction of brain cortical tissue of male WT and 5xFAD mice using LC-MS/MS-based quantitative targeted absolute proteomic analysis (Figure 2, Supplementary Table S3). In order to identify sex-specific changes in protein expression of ABC and SLC transporters in the 5xFAD model, we compared the observed changes in transporter expression in male mice to those in female 5xFAD and WT mice (Figure 3), for which absolute protein expression of transporters was previously reported (Puris et al., 2022c). The comparison of absolute protein expression of transporters for each sex separately by unpaired *t*-test revealed a higher absolute protein expression of ATP-binding cassette subfamily C member 1 (ABCC1) only in male 5xFAD mice compared to sex-matched WT animals (*p* = 0.007) with no differences in female groups. In contrast, absolute protein expressions of glucose transporter 1 (GLUT1) (*p* = 0.01), long-chain fatty acid transport protein 1 (FATP1) (*p* = 0.02) and 4F2hc (*p* = 0.01) were significantly higher in female 5xFAD mice compared to sex-matched WT animals, while no differences in the levels of these proteins were observed in male groups. In addition, a statistically significant increase in absolute protein expression of alanine/serine/cysteine/threonine transporter 1 (ASCT1) was observed in both male (1.5-fold; *p* = 0.02) and female (2.2-fold; *p* = 0.002) 5xFAD mice compared to sex-matched WT animals. No statistically significant differences were observed in the absolute protein expression of ATP-binding cassette subfamily B member 1, ABCB1 (also known as multidrug resistance protein 1, MDR1), ATP-binding cassette subfamily G member 2, ABCG2 (also known as breast cancer resistance protein, BCRP) and large neutral amino acids transporter small subunit 1 (LAT1). The protein expression of membrane marker Na^+^/K^+^-ATPase was similar in all study groups with low intragroup variation indicating comparable enrichment of the extracted membrane fraction between the samples. The comparison of protein expression between female and male WT or 5xFAD mice (Supplementary Figures S1, S2) revealed significantly higher expression of ABCC1, ASCT1, LAT1 and 4F2hc in males compared to females in both phenotypes supporting the fact that the observed discrepancies in transporter expression in 5xFAD mice vs. sex-matched WT controls are sex-specific.

## 4 Discussion

Sex-related differences in AD pathogenesis have been revealed in clinical, pathological, and neuroimaging studies (Guo et al., 2022). However, considering sex in preclinical and clinical studies as an important factor affecting the response to drug candidates has been greatly underappreciated. In the present study, we characterized for the first time a commonly used model of FAD, 5xFAD mice, in terms of sex-dependent changes in transporter protein expression in the brain cortex. Similar to previous studies in 5xFAD mice, here, we observed pronounced sex-related differences in Aβ pathology and inflammation characterized by the presence of activated astrocytes (Oakley et al., 2006; Javonillo et al., 2021; Oblak et al., 2021). Thus, a higher total concentration of Aβ_1-42_ was revealed in female 5xFAD mice compared to male 5xFAD mice. In addition, we observed a greater activation of astrocytes in female 5xFAD mice compared to male 5xFAD mice, as more pronounced changes in gene expression of *Gfap* were observed in female 5xFAD mice vs. sex-matched WT animals than in male mice. The present study did not reveal any sex-specific differences in microglial activation in 5xFAD mice, as shown by gene expression of *Aif1*. Interestingly, gene expression of the pro-inflammatory cytokine *Il1b* was significantly altered only in female 5xFAD mice as compared to sex-matched WT animals. Both Aβ pathology and inflammation, as major hallmarks of AD, can affect expression of transporters in the brain (Kania et al., 2011; Gibson et al., 2012; Shubbar and Penny, 2018; Puris et al., 2021). Sex-related discrepancies in Aβ pathology and inflammation can reflect on various changes in transporter expression in male and female 5xFAD mice.

Among investigated ABC transporters, we revealed sex-specific changes in expression of ABCC1 transporter in the prefrontal cortices of male 5xFAD mice as compared to sex-matched WT controls. Similar to ABCB1 and ABCG2, ABCC1 is expressed in pericytes, astrocytes, neurons, the luminal membrane of the brain capillary endothelial cells, but also in the abluminal site of the BBB (Pereira et al., 2018). Th ABC transporters play important roles in mediating the efflux of drugs and metabolites from the NVU cells and have been shown to contribute to AD progression due to involvement in the clearance of Aβ from the brain (Xiong et al., 2009; Bernstein et al., 2014; Pahnke et al., 2014; Pereira et al., 2018). ABCC1 possesses broad substrate specificity, mediating transport of anionic compounds, including glucuronide, glutathione and sulfate conjugates, as well as different chemotherapeutic agents (Cole, 2014). Although several inhibitors of ABCC1 were identified (Mahringer and Fricker, 2016), the specific clinically relevant ABCC1 inhibitors are not available. Previously, we revealed a significantly increased ABCC1 expression in male TgF344-AD rats compared to sex- and age-matched WT controls, while no changes were observed in female TgF344-AD rats (Puris et al., 2022a). In addition, we have not observed sex-specific changes in ABCB1 and ABCG2 protein expression in TgF344-AD rat model as compared to the respective WT controls (Puris et al., 2022a). Since male 5xFAD mice have demonstrated a significantly lower brain cortical levels of Aβ_1–42_ compared to 5xFAD female mice and a significantly higher protein expression of ABCC1, one can assume that the sex-specific upregulation observed in the male 5xFAD mice may contribute to the slower progression of Aβ pathology in males versus females. Future studies should focus on elucidating the molecular mechanism(s) underlying sex-related alterations in ABCC1 expression and association of ABCC1 expression with the development of Aβ pathology, which can shed light on discrepancies in the AD pathogenesis in men and women. Finally, there is evidence that ABCC1 expression can change throughout the estrous cycle (Jang et al., 2017), which could explain higher intragroup variability in ABCC1 expression in female mice. However, as in the present study, we have not determined the estrus cycle, future studies should address this issue.

In the present study, we observed a sex-specific changes in protein expression of GLUT1 (encoded by *SLC1A2*/*Slc1a2*) with elevated levels of the transporter in female 5xFAD mice as compared to sex-matched WT animals. Two isoforms of GLUT1 are present in the brain: the more glycosylated GLUT1 isoform in the brain microvessels and the less glycosylated in astrocytes. Previously, it was suggested that GLUT1 downregulation aggravates AD progression at the early stages of the disease. Thus, decreased expression of GLUT1 was detected in AD brains using Western blot (Mooradian et al., 1997; Liu et al., 2008). In another quantitative proteomics study, GLUT1 protein expression was not altered in the isolated brain microvessels of AD patients as compared to non-demented individuals (Al-Majdoub et al., 2019). However, in these studies, the protein expression of GLUT1 was investigated in the combined samples from both female and male individuals. Previously, we did not reveal a sex effect on GLUT1 protein expression in the brain prefrontal cortex of TgF344-AD rats as compared to WT controls (Puris et al., 2022a). Interestingly, pro-inflammatory cytokine IL-1β upregulated GLUT1 expression in the brain endothelial cells and astrocytes (Jurcovicova, 2014). Here, we observed upregulated *Il1b* gene expression in the brain cortical tissue of female 5xFAD mice as compared to sex-matched WT controls, providing the evidence that sex-related discrepancies in expression of GLUT1 in the brain cortex of 5xFAD mice can be mediated by IL-1β. As GLUT1 plays important role in AD progression and has been used for drug targeting to the brain (Puris et al., 2022b), understanding of sex-specific changes in expression of this transporter in animal models and AD patients is crucial for development of effective drug treatments.

A significant upregulation of FATP1 protein expression in the brain prefrontal cortical tissue of female 5xFAD mice was observed as compared to corresponding WT controls, while no statistically significant differences were revealed in the brain cortical tissue of male animals. FATP1 is responsible for import of long-chain fatty acids across the membranes of the brain capillary endothelial cells, neurons, and glia (Mitchell et al., 2011; Shawahna et al., 2011; Liu et al., 2017; Ochiai et al., 2017; Puris et al., 2022a; Puris et al., 2023). Recent studies demonstrated potential involvement of FATP1 in AD pathogenesis (Ochiai et al., 2019), while the alterations in the expression and function of this transporter in AD patients have not been studied. Previously, we found a significantly increased protein expression of this transporter in the isolated brain microvessels of TgF344-AD rats as compared to age-matched WT controls (Puris et al., 2022a). However, we did not reveal sex-related changes in FATP1 protein expression in the brain prefrontal cortex of TgF344-AD rats as compared to age- and sex-matched WT controls (Puris et al., 2022a). Further studies should focus on investigation of the sex-specific changes in expression and function of FATP1 in AD brains to elucidate the role of this transporter in AD and provide information on the relevance of animal models to mimic the changes in AD patients.

Another SLC transporter, which demonstrated sex-related changes in protein expression in 5xFAD mice as compared to sex-matched WT animals is a heavy chain 4F2hc (encoded by *SLC3A2*/*Slc3a2*). 4F2hc is a glycoprotein, which is coupled with the light chain functional subunits such as LAT1 and LAT2, mediating the transport of amino acids and considered as targets for drug delivery (Puris et al., 2020; Puris et al., 2022b). The main function of 4F2hc is to localize the light chain functional subunits at the plasma membrane in order to facilitate the transport of their substrates (Verrey et al., 2000). Here, we observed significantly higher brain cortical protein expression of 4F2hc in female 5xFAD mice as compared to female WT mice, while no differences were revealed between male study groups, indicating sex-specific effect of AD phenotype on 4F2hc protein expression. The alterations in expression and function of 4F2hc in AD patients have not been studied. Surprisingly, previously, we observed a significant upregulation of 4F2hc protein expression in male TgF344-AD rats as compared to sex- and age-matched WT animals, but not in female rats (Puris et al., 2022a), demonstrating model-specific discrepancies in transporter expression changes, and highlighting the importance of characterization of animal models for each sex individually. No sex-specific changes in LAT1 (encoded by SLC7A5) protein expression were observed in 5xFAD mice as compared to WT animals. Previous reports have not revealed changes in expression of LAT1 in the NVU of AD patients and animal models (Gynther et al., 2018; Al-Majdoub et al., 2019; Puris et al., 2021; Puris et al., 2022a; Puris et al., 2023).

A significant upregulation was observed in protein expression of ASCT1 in 5xFAD mice of both sexes as compared to age- and sex-matched WT animals with more elevated levels in female 5xFAD mice. ASCT1 (encoded by *SLC1A4*/*Slc1a4*) is a main transporter responsible for neuronal efflux of D-serine, a co-agonist of *N*-methyl-D-aspartate receptors (NMDAR), as well as export of L-serine from astrocytes (Rosenberg et al., 2013; Sason et al., 2017; Kaplan et al., 2018). As D-serine is one of the contributors to cognitive decline in AD and is currently of great interest as a target for AD drug development (Orzylowski et al., 2021), understanding the contribution of ASCT1 to altered D-serine levels in AD brains can open new horizons for the development of drugs targeting this transporter to regulate D-serine brain levels. Recently, we revealed possible involvement of ASCT1 in manifestations of AD via potential regulation of the levels of serine in 8-month-old female 5xFAD mice (Puris et al., 2023). Moreover, we observed elevated protein expression of ASCT1 in both 5xFAD and WT mice after exposure to ultrafine particles, ubiquitous solid components of air pollution (Puris et al., 2022c). In contrast to the results of the present study, we did not observe a sex effect on ASCT1 protein expression in the brain cortex of TgF344-AD rats as compared to WT controls (Puris et al., 2022a). There is lack of information about the expression of ASCT1 in AD brains. Future studies in patients and animal models should reveal if the impact of sex on ASCT1 protein expression in the brain is model-specific or dependent on the stage of AD.

Overall, this study provides novel information about sex-specific changes in transporter protein expression at the brain cortex of 5xFAD mice. The findings of the study can facilitate drug development by providing possible explanation of discrepancies in AD development between male and female. Due to limited knowledge about sex-specific changes in brain transporter expression and function in AD patients, future studies should focus on investigation of such changes in AD patients and comparison to preclinical models. In addition, investigation of the time-course of sex-related changes in transporter expression in the brain can aid in understanding whether the alterations in transporter expression play a role as an initial trigger or resulted from the AD pathology. In summary, the findings presented herein highlights the importance of considering sex during development of transporter-targeting therapies for AD and drug delivery strategies as well as characterization of preclinical AD models to develop effective therapeutic strategies to combat AD.

## Data Availability

The original contributions presented in the study are included in the article/Supplementary Material, further inquiries can be directed to the corresponding author.
