# Supplementary material for: Sex-specific changes in protein expression of membrane transporters in the brain cortex of 5xFAD mouse model of Alzheimer’s disease
Source: Front Pharmacol. 2024 Mar 20;15:1365051. doi: 10.3389/fphar.2024.1365051 (PMC10989684; doi:10.3389/fphar.2024.1365051)
Supplement: Supplementary file 1 [file Table1.DOCX]

Supplementary Material

Sex-specific changes in protein expression of membrane transporters in the brain cortex of 5xFAD mouse model of Alzheimer’s disease

Elena Puris^*^, Liudmila Saveleva, Seppo Auriola, Mikko Gynther, Katja M. Kanninen, Gert Fricker

*** Correspondence:** Corresponding Author: elena.puris@uni-heidelberg.de

# Methods

## Gene expression of inflammation markers

Total RNA extraction from mouse brain cortices was carried out using RNeasy Mini Kit (#74004, Qiagen, Stockach, Germany) in accordance with the manufacturer’s protocol. Synthesis of cDNA was performed using Biozym cDNA synthesis Kit (#331475S, Oldendorf, Germany) in accordance with the manufacturer’s instructions. The PowerUp^TM^SYBR^TM^ Green Master Mix (#A25741, Thermo-Fischer, Waltham, USA) and previously validated gene-specific primers (Tables S1) (Puris et al., 2023) were mixed with the resulted cDNA. Relative gene expression was calculated by normalizing the target gene expression to gene expression of housekeeping gene, i.e., glyceraldehyde‐3‐phosphate dehydrogenase (*Gapdh*), in each sample following the method described previously (Taylor et al., 2019). LightCycler 96 (Roche Diagnostics) was used to perform qRT-PCR analysis. The data were acquired with LightCycler® 96 SW 1.1 software, v. 1.1.0.1320 from Roche Diagnostics, Mannheim, Germany (2011).

## Sample preparation and absolute quantification of transporter protein expression

Briefly, proteins in crude membrane fractions (aliquots of 50 µg of total protein) were solubilized in the buffer containing 7 M guanidine hydrochloride (#G3272, Sigma-Aldrich, St. Louis, MO), 500 mM Tris–HCl (#10812846001, Sigma-Aldrich) (pH 8.5) and 10 mM ethylenediaminetetraacetic acid (EDTA). After that, the proteins were reduced and alkylated with dithiothreitol and iodoacetamide, respectively. Consequently, the samples were precipitated with methanol-chloroform followed by dissolution of obtained precipitates in urea buffer containing in 6 M urea in 0.1 M Tris–HCl (pH 8.5). The samples were diluted 5 times with 100 mM Tris–HCl (pH 8.5) containing internal standard peptides, i.e., stable-isotope labelled quantified peptides (JPT Peptide Technologies GmbH, Berlin, Germany) (Table S2). Protease-Max surfactant (V2072, Promega, Madison, WI, USA) and lysyl endopeptidase (VA1170, Promega, Madison, WI, USA) were added to the samples, which were consequently incubated for 3 h at room temperature, followed by a 16-h tryptic digestion with tosylphenylalanyl chloromethyl ketone-treated trypsin (VA9000, Promega, Madison, WI, USA) (enzyme to substrate ratio of 1:100) at 37 °C. The resulting peptide samples were acidified with formic acid in water 20% (v/v) followed by centrifugation of the samples for 5 min at 14000 × g at 4 °C. The obtained supernatants were injected into an Agilent 1290 Infinity LC (Agilent Technologies, Waldbronn, Germany) system coupled with an Agilent 6495 Triple Quadrupole Mass Spectrometer equipped with an ESI source (Agilent Technologies, Palo Alto, CA, USA). The peptide samples were loaded into Advance Bio Peptide Map column (2.1 × 250 mm; 2.7 μm) for HPLC separation and elution of the peptides as previously described (Puris et al., 2022a). The detection of eluted peptides was carried out by applying the positive ion multiple reaction monitoring (MRM) mode as previously described (Puris et al., 2022a;Puris et al., 2022b;Puris et al., 2023). The acquisition of data was done using the Agilent MassHunter Workstation Acquisition software, Agilent Technologies, Data Acquisition for Triple Quad., version B.03.01. The data processing was performed using Skyline software (version 4.1). The absolute protein expression levels and the limit of quantification were calculated as reported in our previous studies (Puris et al., 2022a;Puris et al., 2022b;Puris et al., 2023).

# Supplementary Figures and Tables

**Supplementary Table S1.** *Primer sequences for qRT-PCR analysis*

| **Gene** | **Forward primer** | **Reverse primer** |
| --- | --- | --- |
| *Gfap* | CACCTACAGGAAATTGCTGGAGG | CCACGATGTTCCTCTTGAGGTG |
| *Aif1* | GGCTTCAAGTTTGGACGGCAGATCCTC | CATGAGCCAAAGCAGGGATTTGCAGGG |
| *Il1b* | GCAACTGTTCCTGAACTCAACT | ATCTTTTGGGGTCCGTCAACT |
| *Gapdh* | CCATGGAGAAGGCTGGGG | CAAAGTTGTCATGGATGACC |

**Supplementary Table S2.** *Probe peptide amino acid sequences and multiple reaction monitoring transitions for the LC-MS/MS-based quantitative targeted absolute proteomics analysis.*

| **Protein/**  **gene name** | **St/IS** | **Unique amino acid sequence** | **Retention time (min)** | **MRM transitions (m/z)** | | | | |
| --- | --- | --- | --- | --- | --- | --- | --- | --- |
|  |  |  |  | **Q1** | **Q3.1** | **Q3.2** | **Q3.3** | **Q3.4** |
| **ABC transporters** | | | | | | | | |
| **ABCB1/*Abcb1*^a^** | St | NTTGALTTR | 8.7 | 467.7 | 719.4 | 618.3 | 561.3 |  |
|  | IS | NTTGALTT**R*** | 8.7 | 472.7 | 729.4 | 628.3 | 517.3 |  |
| **ABCG2/*Abcg2*** | St | SSLLDVLAAR | 27.4 | 522.8 | 757.4 | 644.3 | 529.3 |  |
|  | IS | SSLLDVLAA**R*** | 27.4 | 527.8 | 767.4 | 654.3 | 539.3 |  |
| **ABCC1/*Abcc1*** | St | TPSGNLVNR | 9.9 | 479.2 | 759.4 | 672.3 | 501.3 |  |
|  | IS | TPSGNLVN**R*** | 9.9 | 484.2 | 769.4 | 682.3 | 511.3 |  |
| **SLC transporters** | | | | | | | | |
| **ASCT1/** ***Slc1a4*** | St | ETVDSFLDLLR | 32.2 | 654.3 | 978.5 | 863.5 | 776.5 |  |
|  | IS | ETVDSFLDLL**R*** | 32.2 | 659.3 | 988.5 | 873.5 | 786.5 |  |
| **GLUT1/*Slc2a1*** | St | TFDEIASGFR | 21.1 | 571.7 | 894.4 | 779.4 | 650.4 | 537.3 |
|  | IS | TFDEIASGF**R*** | 21.1 | 576.7 | 904.4 | 789.4 | 660.4 | 547.3 |
| **LAT1/*Slc7a5*** | St | VQDAFAAAK | 12.0 | 460.7 | 693.4 | 578.3 | 507.3 |  |
|  | IS | VQDAFAAA**K*** | 12.0 | 464.8 | 701.4 | 586.3 | 515.3 |  |
| **4F2hc/*Slc3a2*** | St | VAGSPGWVR | 14.6 | 464.7 | 758.4 | 701.4 | 614.3 |  |
|  | IS | VAGSPGWV**R*** | 14.6 | 469.7 | 768.4 | 711.4 | 624.3 |  |
| **FATP1/*Slc27a1*** | St | LLPQVDTTGTFK | 20.5 | 660.4 | 1093.6 | 996.5 | 868.44 | 769.4 |
|  | IS | LLPQVDTTGTF**K*** | 20.5 | 664.4 | 1101.6 | 1004.5 | 876.4 | 777.4 |
| **Plasma membrane marker, abluminal membrane** | | | | | | | | |
| **Na^+^/K^+^–ATPase** | St | AAVPDAVGK | 10.7 | 414.3 | 685.4 | 586.3 | 489.3 |  |
|  | IS | AAVPDAVG**K*** | 10.7 | 418.3 | 693.4 | 594.3 | 497.3 |  |

^a^*Abcb1* refers to both *Abcb1a* and *Abcb1b*

St – standard, IS – internal standard

Bold letter with* denotes labelled arginine (R) or lysine (K) with a stable isotope ^13^C and ^15^N

**Supplementary Table S3.** *Individual levels of protein expression of SLC and ABC transporters (fmol/μg total protein) in crude membrane fraction of the brain cortical tissue of male wild-type (WT) (n = 8) and 5xFAD mice (n = 8).*

| **# animal** | **5xFAD/**  **WT** | **Protein expression (fmol/ug total protein)** | | | | | | | | |
| --- | --- | --- | --- | --- | --- | --- | --- | --- | --- | --- |
|  |  | **ABCB1** | **ABCG2** | **ABCC1** | **ASCT1** | **GLUT1** | **4F2hc** | **LAT1** | **FATP1** | **Na^+^/K^+^-ATPase** |
| LSa-670 | WT | 0.1917 | 0.8475 | 0.1152 | 2.989 | 5.221 | 1.072 | 0.7917 | 1.015 | 46.72 |
| LSa-672 | WT | 0.2583 | 0.8994 | 0.132 | 3.109 | 6.547 | 0.9114 | 0.9699 | 2.715 | 89.18 |
| LSa-679 | WT | 0.1416 | 0.6057 | 0.1887 | 2.361 | 4.817 | 0.8382 | 0.6279 | 0.9255 | 69.26 |
| LSa-680 | WT | 0.2838 | 1.680 | 0.1407 | 3.203 | 9.779 | 1.617 | 0.7656 | 2.403 | 79.81 |
| LSa-682 | WT | 0.1116 | 0.5145 | 0.1506 | 1.445 | 3.810 | 0.5325 | 0.6993 | 0.6993 | 80.27 |
| PKo-614 | WT | 0.2199 | 0.2774 | 0.1447 | 3.559 | 2.622 | 0.7471 | 0.5001 | 0.3743 | 59.24 |
| PKo-620 | WT | 0.2142 | 0.2747 | 0.1341 | 1.5731 | 3.286 | 0.5639 | 0.2832 | 0.3786 | 80.53 |
| PKo-622 | WT | 0.1845 | 0.2502 | 0.1518 | 2.1185 | 3.097 | 0.6236 | 0.3222 | 0.3915 | 84.99 |
| LSa-650 | 5xFAD | 0.2577 | 0.7902 | 0.1734 | 3.886 | 8.107 | 1.315 | 1.091 | 1.556 | 60.98 |
| LSa-660 | 5xFAD | 0.1689 | 0.8505 | 0.1521 | 4.539 | 7.993 | 1.868 | 0.6903 | 1.143 | 62.61 |
| LSa-662 | 5xFAD | 0.2217 | 0.5904 | 0.1668 | 2.663 | 3.766 | 0.7134 | 0.7365 | 0.6300 | 64.86 |
| LSa-678 | 5xFAD | 0.1812 | 0.9192 | 0.156 | 3.356 | 6.376 | 1.237 | 1.125 | 0.9003 | 83.99 |
| PKo-615 | 5xFAD | 0.1034 | 0.2366 | 0.1359 | 2.091 | 2.779 | 0.4605 | 0.402 | 0.3264 | 66.79 |
| PKo-619 | 5xFAD | 0.2324 | 0.3369 | 0.1695 | 3.758 | 3.681 | 1.382 | 0.7407 | 0.4107 | 97.36 |
| PKo-621 | 5xFAD | 0.1013 | 0.2234 | 0.2076 | 4.587 | 2.617 | 1.23 | 0.6432 | 0.3212 | 86.87 |
| Lsa-564 | 5xFAD | 0.1061 | 0.3477 | 0.195 | 5.183 | 3.787 | 1.306 | 0.4974 | 0.4199 | 85.66 |

**
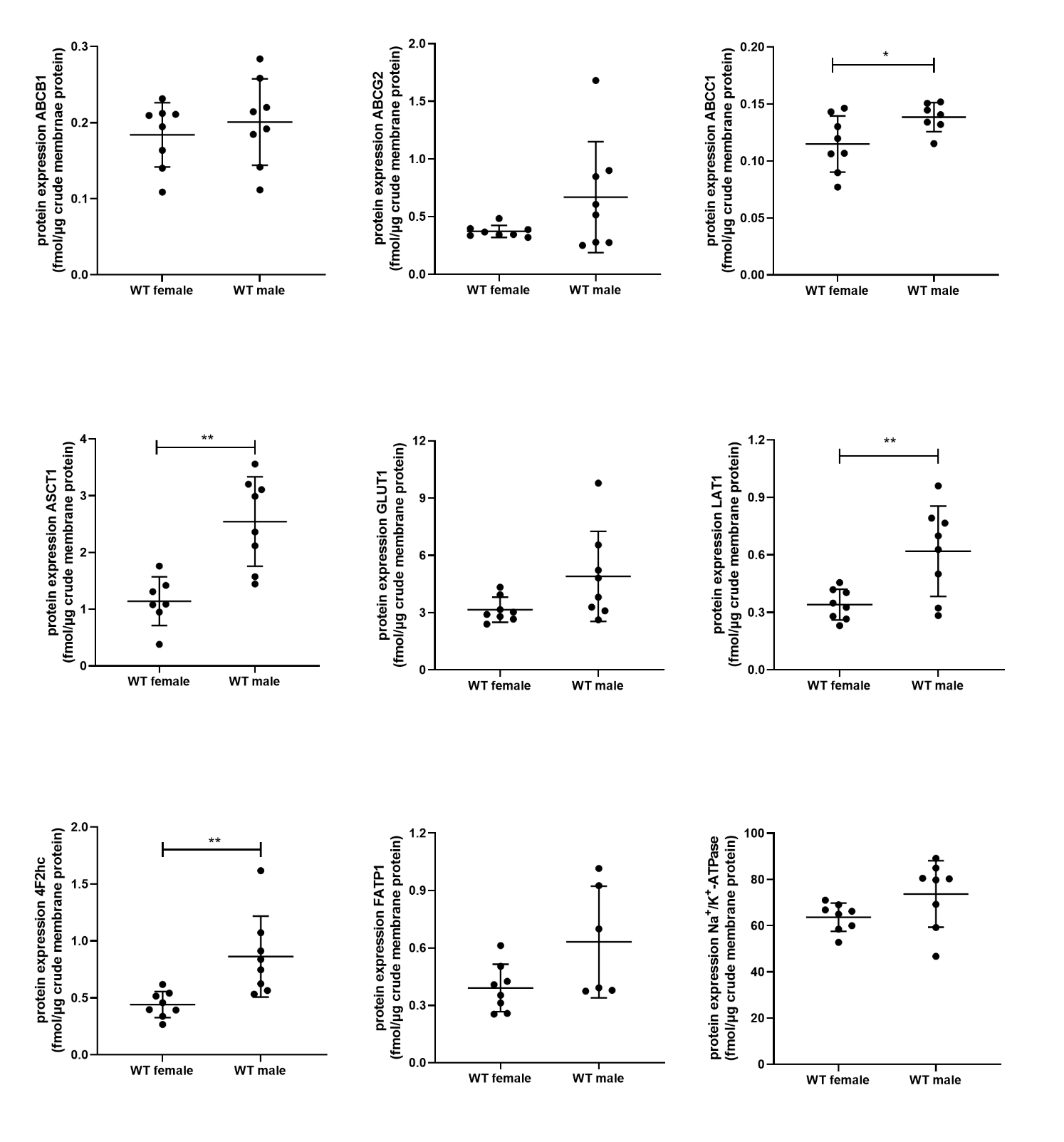
**

**Supplementary Figure S1.** *Comparison of absolute protein expression of ABC and SLC transporters (fmol/μg total protein) in crude membrane fraction of the brain cortical tissue in 7-month-old male (n = 8) and female (n = 8) (Puris et al., 2022b) wild-type (WT) mice. Line represents group mean and whiskers the SD. Statistical significance of changes in protein expression between groups was analyzed by unpaired t-test. Statistically significant differences are marked with asterisks, where * indicates p < 0.05, ** p < 0.01.*

**
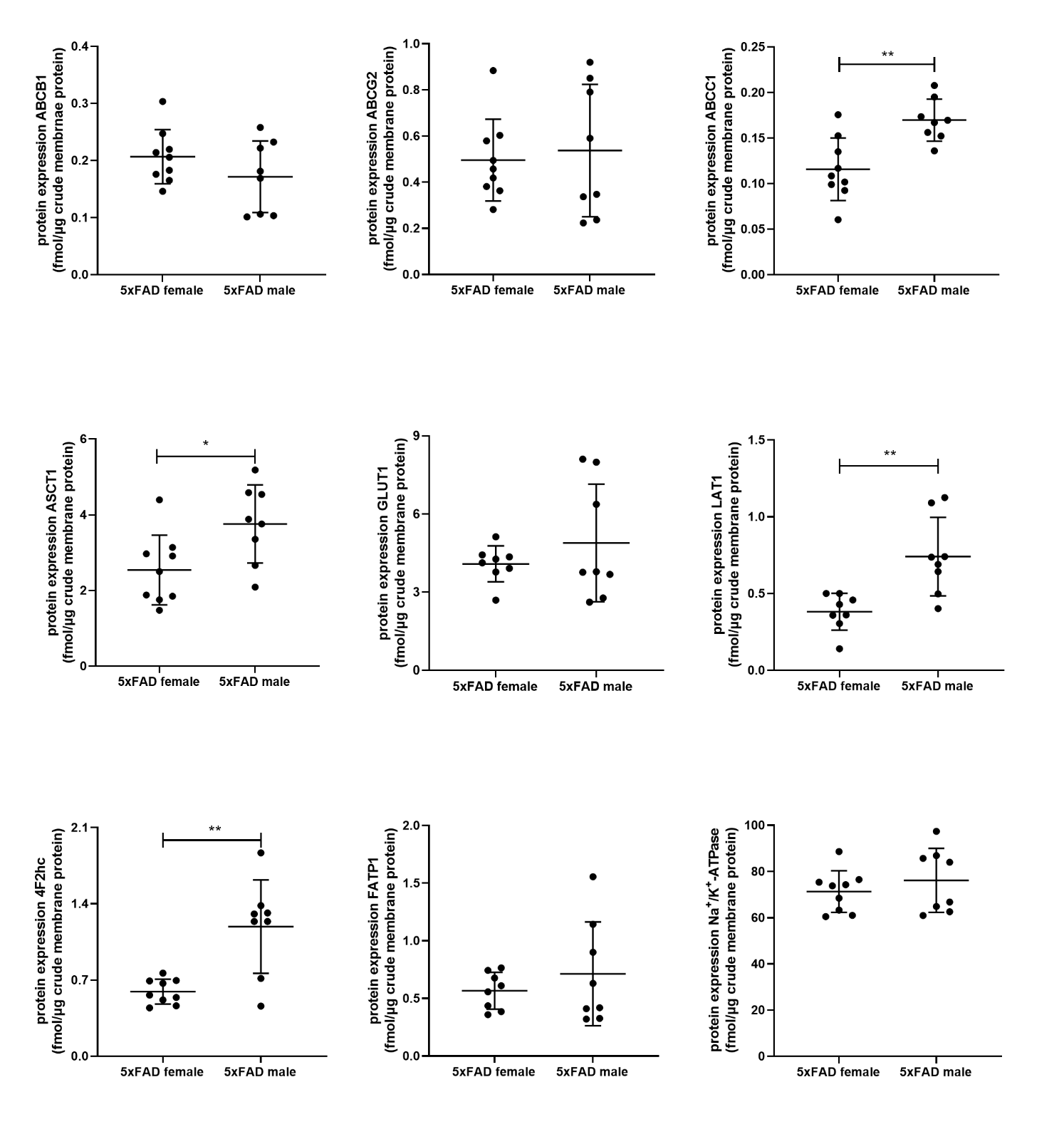
**

**Supplementary Figure S2.** *Comparison of absolute protein expression of ABC and SLC transporters (fmol/μg total protein) in crude membrane fraction of the brain cortical tissue in 7-month-old female (n = 8-9) (Puris et al., 2022b) and male (n = 8) 5xFAD mice. Line represents group mean and whiskers the SD. Statistical significance of changes in protein expression between groups was analyzed by unpaired t-test. Statistically significant differences are marked with asterisks, where * indicates p < 0.05, ** p < 0.01.*

**Results**

## Differences in transporter protein expression in male and female WT or 5xFAD mice

The comparison of transporter expression in the brain cortex of male and female WT mice (Fig. S1) revealed significantly increased protein levels of ABCC1 (*p* = 0.04), ASCT1 (*p* = 0.001), LAT1 (*p* = 0.007) and 4F2hc (*p* = 0.006) in male mice as compared to female animals. Similarly, the protein expression of the same ABCC1 (*p* = 0.002), ASCT1 (*p* = 0.02), LAT1 (*p* = 0.003) and 4F2hc (*p* = 0.001) was higher in male 5xFAD mice as compared to female 5xFAD mice. These findings indicate the overall higher expression of the mentioned above transporters in both phenotypes in male mice compared to female animals.

**References**

Puris, E., Auriola, S., Petralla, S., Hartman, R., Gynther, M., De Lange, E.C.M., and Fricker, G. (2022a). Altered protein expression of membrane transporters in isolated cerebral microvessels and brain cortex of a rat Alzheimer's disease model. *Neurobiol Dis* 169**,** 105741.

Puris, E., Saveleva, L., De Sousa Maciel, I., Kanninen, K.M., Auriola, S., and Fricker, G. (2023). Protein Expression of Amino Acid Transporters Is Altered in Isolated Cerebral Microvessels of 5xFAD Mouse Model of Alzheimer's Disease. *Mol Neurobiol* 60**,** 732-748.

Puris, E., Saveleva, L., Gorova, V., Vartiainen, P., Kortelainen, M., Lamberg, H., Sippula, O., Malm, T., Jalava, P.I., Auriola, S., Fricker, G., and Kanninen, K.M. (2022b). Air pollution exposure increases ABCB1 and ASCT1 transporter levels in mouse cortex. *Environ Toxicol Pharmacol* 96**,** 104003.

Taylor, S.C., Nadeau, K., Abbasi, M., Lachance, C., Nguyen, M., and Fenrich, J. (2019). The Ultimate qPCR Experiment: Producing Publication Quality, Reproducible Data the First Time. *Trends Biotechnol* 37**,** 761-774.
